# The Empire State Express

**Published:** 1891-12

**Authors:** 


### THE EMPIRE STATE EXPRESS.

WITHIN the past month the New York Central & Hudson River Railroad has once more astonished the traveling public by placing at its service the fastest train in the world, which it has appropriately named the Empire State Express. The running time of this train between New York and Buffalo, is eight hours and forty minutes, which gives an average of about fifty-two and a half miles an hour, including stops, a feat that has been accomplished by no other railway in the world, and it is safe to say that it is utterly impossible for any other railroad to approach this speed for so long a distance. Think of it! A passenger may take his breakfast in New York, his luncheon en route, spend three hours in Buffalo and dine at six o'clock leisurely; then take the 8.35 Chicago Limited, and arrive in the city of the Fair next morning to breakfast.

But this is not all; he may take his breakfast in New York, and his dinner at Niagara Falls, arriving at the latter station at 7.20 in the evening. Besides this he may traverse one of the most beautiful regions in the world; along the Hudson he will see the rugged, high banks of the palisades, then the picturesque margin of the river on both sides, dotted here and there with thrifty cities and towns, and finally, before noon, reach Albany, the capital city of the Empire State, which is famous in its old Dutch history; then he bowls along the Mohawk valley for 150 miles at the rate of a mile a minute through one of the most fertile, picturesque, and historic regions in our whole country; he rides through populous cities of 100,000 to 150,000 inhabitants, passes through smart towns of 5,000 to 10,000, each noted for manufacturing and other business enterprises, observes en route the finest agricultural region of the world, and finally reaches Buffalo, not very tired, but just a little hungry, and ready to accomplish a day's work in a couple of hours, and go back by the Buffalo special at 7.30. There is no extra fare charged on this train, and it is only one more and greater convenience that the New York Central, ever ready to oblige its patrons, has placed within the reach of the traveler. There are eleven other splendidly equipped trains daily traversing the State from one end to the other, running over the great four-track road, which has been truly pronounced America's greatest railway.

The equipment of the Empire State Express is the most superb that can be obtained, and consists of one combination buffet smoking-car, two standard New York Central coaches, and one Wagner buffet drawing-room car, while the entire train is heated by steam, lighted by gas, and possesses all the modern conveniences and luxuries of palatial traveling. Its cuisine supplies the choicest edibles, from which a luncheon is served, and where choice wines and cigars can be obtained at all hours. We hope that this innovation of the New York Central in providing a train with such speed will induce the travelers to ride more in the daytime than heretofore. We are of the opinion that much ill-health is produced by traveling at night, and shall have something to say later upon this subject in the JOURNAL. Our chief point has been especially to call the attention of our readers to the New York Central as offering one of the cheapest, speediest, safest, and most healthful methods of covering the distance between New York and Buffalo.

Physicians are interested in these questions, hence we allude to the subject at some length.

